# 5,8-Dibromo-15,18-dimeth­oxy-2,11-dithia­[3.3]paracyclo­phane

**DOI:** 10.1107/S1600536810029053

**Published:** 2010-07-31

**Authors:** Guojun Jin, Yinghui Lu

**Affiliations:** a Key Laboratory of Pesticides and Chemical Biology of the Ministry of Education, College of Chemistry, Central China Normal University, Wuhan 430079, People’s Republic of China

## Abstract

In the title compound [systematic name: 1^2^,1^5^-dibromo-5^2^,5^5^-dimethoxy-2,7-dithia-1,5(1,4)-dibenzenaoctaphane], C_18_H_18_Br_2_O_2_S_2_, the dihedral angle between the aromatic rings is 0.6 (2)° and their centroid separation is 3.251 (2) Å, indicating that a trans-annular π–π interaction occurs. The dimeth­oxy and dibromo substituents are located at crossed positions because of the electronic and the steric nature of the substituents.

## Related literature

For the preparation of the title compound, see: Kay & Baek (1997 ▶); Xu *et al.* (2008 ▶). For paracyclo­phane and its derivatives, see: Clément *et al.* (2009 ▶); Wang *et al.* (2006 ▶); Yamamoto *et al.* (1997 ▶). For studies on the benzene dimer of [2.2]paracyclo­phane, see: Ball *et al.* (2004 ▶); Dahmen & Bräse (2002 ▶); Rowlands (2008 ▶); Valentini *et al.* (2008 ▶). For studies of [3.3]paracyclo­phane, see: Wang *et al.* (2004 ▶).
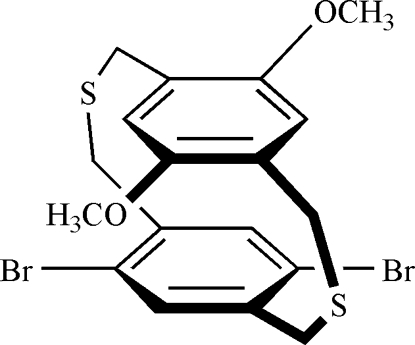


## Experimental

### 

#### Crystal data

C_18_H_18_Br_2_O_2_S_2_
*M* *_r_* = 490.26
Monoclinic, 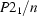

*a* = 8.9576 (8) Å
*b* = 16.2291 (14) Å
*c* = 13.0251 (11) Å
β = 103.240 (1)°
*V* = 1843.2 (3) Å^3^
*Z* = 4
Mo *K*α radiation
μ = 4.63 mm^−1^
*T* = 298 K
0.16 × 0.12 × 0.10 mm

#### Data collection

Bruker SMART CCD area-detector diffractometer
12800 measured reflections
4475 independent reflections
2812 reflections with *I* > 2σ(*I*)
*R*_int_ = 0.126

#### Refinement

*R*[*F* ^2^ > 2σ(*F* ^2^)] = 0.059
*wR*(*F* ^2^) = 0.151
*S* = 0.95
4475 reflections
219 parameters
H-atom parameters constrained
Δρ_max_ = 1.00 e Å^−3^
Δρ_min_ = −1.27 e Å^−3^

### 

Data collection: *SMART* (Bruker, 1997 ▶); cell refinement: *SAINT* (Bruker, 1999 ▶); data reduction: *SAINT*; program(s) used to solve structure: *SHELXS97* (Sheldrick, 2008 ▶); program(s) used to refine structure: *SHELXL97* (Sheldrick, 2008 ▶); molecular graphics: *SHELXTL* (Sheldrick, 2008 ▶); software used to prepare material for publication: *SHELXL97*.

## Supplementary Material

Crystal structure: contains datablocks I, New_Global_Publ_Block. DOI: 10.1107/S1600536810029053/si2274sup1.cif

Structure factors: contains datablocks I. DOI: 10.1107/S1600536810029053/si2274Isup2.hkl

Additional supplementary materials:  crystallographic information; 3D view; checkCIF report

## Figures and Tables

**Fig. 1.**
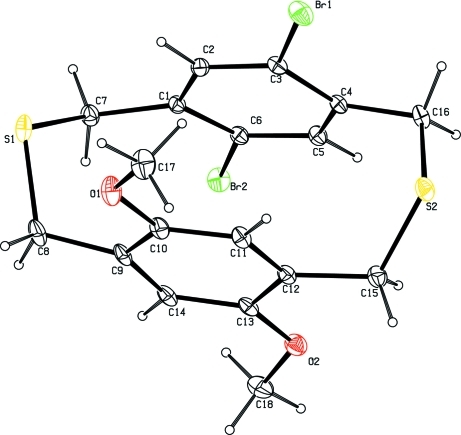
Molecular structure of the tite compound with displacement ellipsoids drawn at the 30% probability level.

## supplementary crystallographic information

### Comment

Various studies on the benzene dimer of [2.2]paracyclophane have focused on the face-to-face stacking (Rowlands, 2008),it is known to play a significant role in chiral catalysis (Dahmen & Bräse, 2002), molecular electronics (Ball *et al.*,2004), and organic solar cells (Valentini *et al.*,2008). However, the [3.3]paracyclophane have received less attention (Wang *et al.*2004). In our research, we have synthetized a series of novel dithia[3.3]paracyclophane. The inter plane distance of the two benzene rings of 3.251Å is less than the normal packing distance of aromatic rings in organic aromatic molecules(3.4°), thus suggesting probable transannular π-π interaction.

For the preparation of the title compound, see: Kay & Baek (1997); Xu *et al.* (2008); for the paracyclophanes and its derivatives, see: Clément *et al.* (2009); Wang *et al.* (2006); Yamamoto *et al.* (1997).

### Experimental

A solution with equimolar amounts of 2,5-dibromo-1,4-bis(mercaptomethyl)benzene (3.26 g,10 mmol) and 1,4-dibromomethyl-2,5-dimethoxybenzene(3.22 g,10 mmol) in degassed THF(500 mL) was added dropwise under N_2_ over 12 h to a refluxing solution of potassium carbonate(6.9 g,50 mmol) in EtOH(1.5*L*). After an additional 2 h at the reflux temperature (353 K), the mixture was cooled and the solvent were removed. The resulting residue was treated with CH_2_Cl_2_(500 mL) and water(500 mL). The organic phase was separated, the aqueous extracted with CH_2_Cl_2_ three times. The combined organic layers was dried over Na_2_SO_4_,then the solvent was removed, and the resulting solid was chromatographed on silica gel using CH_2_Cl_2_/petroleum ether(1:1,*v*/*v*) as eluent. Colourless single crystals of the title compound suitable for X-ray diffraction were obtained by slow evaporation of a dichloromethane/n-hexane(1:30) solution over a period of 5 days.

### Refinement

Hydrogen atoms were placed in calculated positions and refined using a riding model with C—H = 0.93 - 0.97 Å and *U*_iso_(H) = 1.2*U*_eq_(C-H, CH_2_), 1.5*U*_eq_(CH_3_).

### Crystal data

**Table:**

C_18_H_18_Br_2_O_2_S_2_	*F*(000) = 976
*M**_r_* = 490.26	*D*_x_ = 1.767 Mg m^−^^3^
Monoclinic, *P*2_1_/*n*	Mo *K*α radiation, λ = 0.71073 Å
Hall symbol: -P 2yn	Cell parameters from 3885 reflections
*a* = 8.9576 (8) Å	θ = 2.5–26.9°
*b* = 16.2291 (14) Å	µ = 4.63 mm^−^^1^
*c* = 13.0251 (11) Å	*T* = 298 K
β = 103.240 (1)°	Block, colorless
*V* = 1843.2 (3) Å^3^	0.16 × 0.12 × 0.10 mm
*Z* = 4	

### Data collection

**Table:**

Bruker SMART CCD area detector diffractometer	2812 reflections with *I* > 2σ(*I*)
Radiation source: fine-focus sealed tube	*R*_int_ = 0.126
graphite	θ_max_ = 28.3°, θ_min_ = 2.0°
phi and ω scans	*h* = −11→11
12800 measured reflections	*k* = −19→21
4475 independent reflections	*l* = −15→17

### Refinement

**Table:**

Refinement on *F*^2^	Primary atom site location: structure-invariant direct methods
Least-squares matrix: full	Secondary atom site location: difference Fourier map
*R*[*F*^2^ > 2σ(*F*^2^)] = 0.059	Hydrogen site location: inferred from neighbouring sites
*wR*(*F*^2^) = 0.151	H-atom parameters constrained
*S* = 0.95	*w* = 1/[σ^2^(*F*_o_^2^) + (0.0753*P*)^2^] where *P* = (*F*_o_^2^ + 2*F*_c_^2^)/3
4475 reflections	(Δ/σ)_max_ = 0.001
219 parameters	Δρ_max_ = 1.00 e Å^−^^3^
0 restraints	Δρ_min_ = −1.27 e Å^−^^3^

### Special details

**Table:**

Geometry. All e.s.d.'s (except the e.s.d. in the dihedral angle between two l.s. planes) are estimated using the full covariance matrix. The cell e.s.d.'s are taken into account individually in the estimation of e.s.d.'s in distances, angles and torsion angles; correlations between e.s.d.'s in cell parameters are only used when they are defined by crystal symmetry. An approximate (isotropic) treatment of cell e.s.d.'s is used for estimating e.s.d.'s involving l.s. planes.
Refinement. Refinement of *F*^2^ against ALL reflections. The weighted *R*-factor *wR* and goodness of fit *S* are based on *F*^2^, conventional *R*-factors *R* are based on *F*, with *F* set to zero for negative *F*^2^. The threshold expression of *F*^2^ > σ(*F*^2^) is used only for calculating *R*-factors(gt) *etc*. and is not relevant to the choice of reflections for refinement. *R*-factors based on *F*^2^ are statistically about twice as large as those based on *F*, and *R*- factors based on ALL data will be even larger.

### Fractional atomic coordinates and isotropic or equivalent isotropic displacement parameters (Å^2^)

**Table:**

	*x*	*y*	*z*	*U*_iso_*/*U*_eq_	
Br1	0.56512 (6)	0.00221 (3)	0.68306 (4)	0.0672 (2)	
Br2	1.04343 (6)	0.17844 (3)	0.44267 (4)	0.05796 (19)	
C1	0.9651 (5)	0.0721 (2)	0.6001 (3)	0.0383 (9)	
C2	0.8556 (5)	0.0352 (2)	0.6428 (3)	0.0414 (9)	
H2	0.8858	−0.0067	0.6919	0.050*	
C3	0.7037 (5)	0.0569 (2)	0.6166 (3)	0.0421 (9)	
C4	0.6510 (5)	0.1210 (2)	0.5448 (3)	0.0426 (9)	
C5	0.7568 (5)	0.1535 (2)	0.4937 (3)	0.0419 (9)	
H5	0.7248	0.1924	0.4408	0.050*	
C6	0.9099 (5)	0.1296 (2)	0.5193 (3)	0.0386 (9)	
C7	1.1351 (5)	0.0552 (3)	0.6408 (3)	0.0478 (10)	
H7A	1.1921	0.1026	0.6250	0.057*	
H7B	1.1630	0.0085	0.6027	0.057*	
C8	1.2053 (5)	0.1368 (3)	0.8357 (3)	0.0603 (13)	
H8A	1.2330	0.1323	0.9120	0.072*	
H8B	1.2865	0.1669	0.8140	0.072*	
C9	1.0584 (5)	0.1853 (3)	0.8039 (3)	0.0483 (11)	
C10	0.9312 (5)	0.1636 (3)	0.8413 (3)	0.0466 (10)	
C11	0.7901 (5)	0.1997 (3)	0.7979 (3)	0.0461 (10)	
H11	0.7041	0.1842	0.8220	0.055*	
C12	0.7759 (5)	0.2590 (3)	0.7186 (3)	0.0423 (9)	
C13	0.9054 (5)	0.2850 (3)	0.6891 (3)	0.0447 (10)	
C14	1.0460 (5)	0.2476 (3)	0.7292 (3)	0.0480 (10)	
H14	1.1321	0.2642	0.7061	0.058*	
C15	0.6193 (5)	0.2917 (3)	0.6623 (4)	0.0519 (11)	
H15A	0.6279	0.3141	0.5948	0.062*	
H15B	0.5928	0.3368	0.7035	0.062*	
C16	0.4926 (5)	0.1575 (3)	0.5276 (4)	0.0531 (11)	
H16A	0.4179	0.1132	0.5143	0.064*	
H16B	0.4745	0.1922	0.4654	0.064*	
C17	0.8336 (7)	0.0803 (4)	0.9594 (4)	0.0767 (16)	
H17A	0.7924	0.1276	0.9874	0.115*	
H17B	0.8688	0.0411	1.0149	0.115*	
H17C	0.7553	0.0555	0.9054	0.115*	
C18	1.0126 (6)	0.3817 (3)	0.5888 (4)	0.0703 (14)	
H18A	1.0703	0.4095	0.6503	0.106*	
H18B	0.9807	0.4208	0.5328	0.106*	
H18C	1.0756	0.3402	0.5673	0.106*	
O1	0.9536 (4)	0.1039 (2)	0.9173 (2)	0.0666 (9)	
O2	0.8860 (4)	0.34533 (19)	0.6119 (2)	0.0584 (8)	
S1	1.19297 (14)	0.03433 (8)	0.77929 (9)	0.0601 (3)	
S2	0.46307 (13)	0.21831 (8)	0.63885 (10)	0.0559 (3)	

### Atomic displacement parameters (Å^2^)

**Table:**

	*U*^11^	*U*^22^	*U*^33^	*U*^12^	*U*^13^	*U*^23^
Br1	0.0566 (3)	0.0782 (4)	0.0670 (4)	−0.0235 (2)	0.0142 (3)	0.0087 (2)
Br2	0.0558 (3)	0.0679 (3)	0.0542 (3)	−0.0029 (2)	0.0209 (2)	0.0073 (2)
C1	0.038 (2)	0.041 (2)	0.0332 (19)	0.0023 (16)	0.0015 (15)	−0.0064 (16)
C2	0.046 (2)	0.039 (2)	0.036 (2)	−0.0006 (18)	0.0028 (17)	0.0016 (17)
C3	0.043 (2)	0.046 (2)	0.036 (2)	−0.0103 (18)	0.0058 (17)	−0.0035 (17)
C4	0.039 (2)	0.048 (2)	0.035 (2)	−0.0046 (18)	−0.0025 (16)	−0.0061 (17)
C5	0.045 (2)	0.050 (2)	0.0287 (19)	−0.0017 (18)	0.0036 (16)	0.0002 (17)
C6	0.041 (2)	0.045 (2)	0.0292 (18)	−0.0044 (17)	0.0055 (16)	−0.0023 (16)
C7	0.043 (2)	0.059 (3)	0.040 (2)	0.005 (2)	0.0048 (18)	−0.0066 (19)
C8	0.041 (3)	0.097 (4)	0.036 (2)	0.002 (2)	−0.0063 (18)	−0.010 (2)
C9	0.036 (2)	0.072 (3)	0.032 (2)	−0.005 (2)	−0.0033 (17)	−0.018 (2)
C10	0.049 (3)	0.064 (3)	0.0253 (19)	0.000 (2)	0.0041 (17)	−0.0072 (18)
C11	0.037 (2)	0.064 (3)	0.039 (2)	−0.0033 (19)	0.0114 (17)	−0.0076 (19)
C12	0.036 (2)	0.051 (2)	0.038 (2)	−0.0044 (18)	0.0059 (16)	−0.0099 (18)
C13	0.043 (2)	0.054 (2)	0.034 (2)	−0.0112 (19)	0.0024 (17)	−0.0136 (18)
C14	0.037 (2)	0.068 (3)	0.037 (2)	−0.011 (2)	0.0034 (16)	−0.018 (2)
C15	0.040 (2)	0.059 (3)	0.056 (3)	−0.003 (2)	0.010 (2)	0.002 (2)
C16	0.032 (2)	0.072 (3)	0.050 (2)	−0.003 (2)	−0.0010 (18)	−0.001 (2)
C17	0.077 (4)	0.100 (4)	0.054 (3)	0.002 (3)	0.017 (3)	0.027 (3)
C18	0.068 (3)	0.069 (3)	0.072 (3)	−0.023 (3)	0.014 (3)	−0.004 (3)
O1	0.055 (2)	0.102 (3)	0.0419 (17)	0.0117 (19)	0.0104 (15)	0.0129 (17)
O2	0.056 (2)	0.0589 (18)	0.058 (2)	−0.0108 (16)	0.0077 (15)	0.0047 (15)
S1	0.0459 (7)	0.0797 (8)	0.0499 (7)	0.0193 (6)	0.0010 (5)	0.0161 (6)
S2	0.0312 (6)	0.0714 (8)	0.0654 (8)	−0.0008 (5)	0.0113 (5)	−0.0028 (6)

### Geometric parameters (Å, °)

**Table:**

Br1—C3	1.889 (4)	C10—C11	1.390 (6)
Br2—C6	1.898 (4)	C11—C12	1.397 (6)
C1—C2	1.372 (6)	C11—H11	0.9300
C1—C6	1.408 (5)	C12—C13	1.369 (6)
C1—C7	1.518 (6)	C12—C15	1.521 (6)
C2—C3	1.371 (6)	C13—O2	1.386 (5)
C2—H2	0.9300	C13—C14	1.388 (6)
C3—C4	1.407 (5)	C14—H14	0.9300
C4—C5	1.382 (6)	C15—S2	1.809 (4)
C4—C16	1.506 (6)	C15—H15A	0.9700
C5—C6	1.390 (6)	C15—H15B	0.9700
C5—H5	0.9300	C16—S2	1.822 (5)
C7—S1	1.792 (4)	C16—H16A	0.9700
C7—H7A	0.9700	C16—H16B	0.9700
C7—H7B	0.9700	C17—O1	1.369 (6)
C8—C9	1.508 (6)	C17—H17A	0.9600
C8—S1	1.812 (5)	C17—H17B	0.9600
C8—H8A	0.9700	C17—H17C	0.9600
C8—H8B	0.9700	C18—O2	1.372 (6)
C9—C10	1.384 (6)	C18—H18A	0.9600
C9—C14	1.389 (6)	C18—H18B	0.9600
C10—O1	1.368 (5)	C18—H18C	0.9600
			
C2—C1—C6	115.5 (4)	C12—C11—H11	119.6
C2—C1—C7	122.2 (4)	C13—C12—C11	118.7 (4)
C6—C1—C7	122.2 (4)	C13—C12—C15	120.3 (4)
C3—C2—C1	123.3 (4)	C11—C12—C15	120.9 (4)
C3—C2—H2	118.4	C12—C13—O2	116.6 (4)
C1—C2—H2	118.4	C12—C13—C14	120.9 (4)
C2—C3—C4	121.2 (4)	O2—C13—C14	122.3 (4)
C2—C3—Br1	119.0 (3)	C13—C14—C9	120.1 (4)
C4—C3—Br1	119.8 (3)	C13—C14—H14	119.9
C5—C4—C3	116.1 (4)	C9—C14—H14	119.9
C5—C4—C16	120.4 (4)	C12—C15—S2	116.4 (3)
C3—C4—C16	123.4 (4)	C12—C15—H15A	108.2
C4—C5—C6	121.7 (4)	S2—C15—H15A	108.2
C4—C5—H5	119.1	C12—C15—H15B	108.2
C6—C5—H5	119.1	S2—C15—H15B	108.2
C5—C6—C1	121.5 (4)	H15A—C15—H15B	107.3
C5—C6—Br2	117.6 (3)	C4—C16—S2	113.5 (3)
C1—C6—Br2	120.9 (3)	C4—C16—H16A	108.9
C1—C7—S1	114.9 (3)	S2—C16—H16A	108.9
C1—C7—H7A	108.5	C4—C16—H16B	108.9
S1—C7—H7A	108.5	S2—C16—H16B	108.9
C1—C7—H7B	108.5	H16A—C16—H16B	107.7
S1—C7—H7B	108.5	O1—C17—H17A	109.5
H7A—C7—H7B	107.5	O1—C17—H17B	109.5
C9—C8—S1	113.5 (3)	H17A—C17—H17B	109.5
C9—C8—H8A	108.9	O1—C17—H17C	109.5
S1—C8—H8A	108.9	H17A—C17—H17C	109.5
C9—C8—H8B	108.9	H17B—C17—H17C	109.5
S1—C8—H8B	108.9	O2—C18—H18A	109.5
H8A—C8—H8B	107.7	O2—C18—H18B	109.5
C10—C9—C14	119.4 (4)	H18A—C18—H18B	109.5
C10—C9—C8	120.5 (4)	O2—C18—H18C	109.5
C14—C9—C8	119.8 (4)	H18A—C18—H18C	109.5
O1—C10—C9	116.0 (4)	H18B—C18—H18C	109.5
O1—C10—C11	124.2 (4)	C10—O1—C17	119.3 (4)
C9—C10—C11	119.7 (4)	C18—O2—C13	119.4 (4)
C10—C11—C12	120.7 (4)	C7—S1—C8	102.2 (2)
C10—C11—H11	119.6	C15—S2—C16	104.1 (2)
			
C6—C1—C2—C3	−5.6 (6)	O1—C10—C11—C12	−178.4 (4)
C7—C1—C2—C3	171.5 (4)	C9—C10—C11—C12	−1.5 (6)
C1—C2—C3—C4	−1.6 (6)	C10—C11—C12—C13	−4.2 (6)
C1—C2—C3—Br1	−179.0 (3)	C10—C11—C12—C15	173.1 (4)
C2—C3—C4—C5	7.4 (6)	C11—C12—C13—O2	−178.4 (4)
Br1—C3—C4—C5	−175.2 (3)	C15—C12—C13—O2	4.2 (5)
C2—C3—C4—C16	−168.8 (4)	C11—C12—C13—C14	6.4 (6)
Br1—C3—C4—C16	8.6 (5)	C15—C12—C13—C14	−171.0 (4)
C3—C4—C5—C6	−6.0 (6)	C12—C13—C14—C9	−2.8 (6)
C16—C4—C5—C6	170.4 (4)	O2—C13—C14—C9	−177.7 (3)
C4—C5—C6—C1	−1.2 (6)	C10—C9—C14—C13	−3.0 (6)
C4—C5—C6—Br2	179.7 (3)	C8—C9—C14—C13	171.4 (4)
C2—C1—C6—C5	7.0 (5)	C13—C12—C15—S2	141.2 (3)
C7—C1—C6—C5	−170.2 (4)	C11—C12—C15—S2	−36.0 (5)
C2—C1—C6—Br2	−174.0 (3)	C5—C4—C16—S2	−105.9 (4)
C7—C1—C6—Br2	8.8 (5)	C3—C4—C16—S2	70.2 (5)
C2—C1—C7—S1	−32.5 (5)	C9—C10—O1—C17	178.9 (4)
C6—C1—C7—S1	144.4 (3)	C11—C10—O1—C17	−4.1 (7)
S1—C8—C9—C10	69.9 (5)	C12—C13—O2—C18	171.4 (4)
S1—C8—C9—C14	−104.4 (4)	C14—C13—O2—C18	−13.5 (6)
C14—C9—C10—O1	−177.8 (4)	C1—C7—S1—C8	−80.3 (4)
C8—C9—C10—O1	7.9 (6)	C9—C8—S1—C7	56.6 (4)
C14—C9—C10—C11	5.1 (6)	C12—C15—S2—C16	−76.7 (4)
C8—C9—C10—C11	−169.2 (4)	C4—C16—S2—C15	53.5 (4)
